# Brain self-regulation in criminal psychopaths

**DOI:** 10.1038/srep09426

**Published:** 2015-03-24

**Authors:** Lilian Konicar, Ralf Veit, Hedwig Eisenbarth, Beatrix Barth, Paolo Tonin, Ute Strehl, Niels Birbaumer

**Affiliations:** 1 Institute of Medical Psychology and Behavioural Neurobiology, Eberhard-Karls-University of Tübingen, Tübingen, Germany; 2 International Centre for Ethics in the Science and Humanities, Eberhard-Karls-University of Tübingen, Tübingen, Germany; 3 Department of Forensic Psychiatry and Psychotherapy, University of Regensburg, Regensburg, Germany; 4 Department of Psychology, University of Southampton, Southampton, United Kingdom; 5 Ospedale San Camillo, Istituto di Ricovero e Cura a Carattere Scientifico, Venezia, Italy

## Abstract

Psychopathic individuals are characterized by impaired affective processing, impulsivity, sensation-seeking, poor planning skills and heightened aggressiveness with poor self-regulation. Based on brain self-regulation studies using neurofeedback of Slow Cortical Potentials (SCPs) in disorders associated with a dysregulation of cortical activity thresholds and evidence of deficient cortical functioning in psychopathy, a neurobiological approach seems to be promising in the treatment of psychopathy. The results of our intensive brain regulation intervention demonstrate, that psychopathic offenders are able to gain control of their brain excitability over fronto-central brain areas. After SCP self-regulation training, we observed reduced aggression, impulsivity and behavioral approach tendencies, as well as improvements in behavioral-inhibition and increased cortical sensitivity for error-processing. This study demonstrates improvements on the neurophysiological, behavioral and subjective level in severe psychopathic offenders after SCP-neurofeedback training and could constitute a novel neurobiologically-based treatment for a seemingly change-resistant group of criminal psychopaths.

Psychopathy is a personality construct accompanied by a spectrum of changes in affective processing, poor planning skills, impulsivity, disinhibition, sensation-seeking, aggressiveness and a lack of behavioral control, often yielding in antisocial, criminal behavior^1^.

The deficient behavioral control and heightened aggressive approach behavior have been linked to an increased behavioral activation system, sensitive to reward/non punishment^2^,^3^,^4^,^5^,^6^,^7^. Emotional and cognitive abnormalities in individuals with psychopathic and antisocial characteristics were repeatedly demonstrated by findings of a diminished reactivity to aversive and negative emotional stimuli and incomplete or absent anticipatory avoidance learning, reflected in a diminished startle potentiation or the absent skin conductance responses in anticipation of punishment^8^,^9^,^10^,^11^,^12^. The reduced peripheral-physiological responses found in these learning paradigms are in line with studies reporting aberrant cognitive processing and reduced cortical activity after errors in externalizing pathologies like psychopathy and Attention-Deficit-/Hyperactivity Disorder (ADHD)^13^,^14^,^15^,^16^,^17^,^18^,^19^,^20^. The frequently observed EEG abnormality related to violence and antisocial behavior: a higher ratio of low frequencies^21^,^22^,^23^,^24^ (similar to findings of frontal hypo-activation in ADHD) is supported by functional and structural imaging studies, revealing prefrontal dysfunction in psychopathy (for review see Ref. 25). Together with findings of psychopathic abnormal functioning in subcortical regions, a growing body of evidence associates psychopathy with aberrant activity of prefrontal-limbic circuits (including prefrontal cortex, anterior cingulate cortex, insula and amygdala)^25^,^26^,^27^ and the connections between these regions^28^,^29^.

The cognitive and behavioral problems of psychopathy such as poor anticipatory planning, self-regulation and formation of stable expectancies regulated by those pre-frontal-limbic circuits are tightly correlated with the development of slow cortical potentials (SCP) at the cortical surface^12^,^30^,^31^,^32^. SCPs consist of electrically negative or positive polarized slow potentials recorded with EEG amplifiers characterized by long time constants. Electrical negative SCP shifts indicate excitatory mobilization, while electrical positive SCP shifts indicate a reduction of neuronal preparation. SCPs reflect changes in the activity level of the upper cortical layers and regulate thresholds of excitability in cortical cell assemblies^30^. A large body of psychophysiological literature (for review see Ref. 30) documented the relationship of expectancy formation, self-regulation and fronto-central SCP-amplitudes. Preliminary evidence has demonstrated deviant SCP-amplitudes in criminal psychopaths^31^,^32^. The anterior cingulate cortex (ACC), densely connected to the fronto-central cortex for the integration of attentional, affective, and visceral information^33^ has not only been associated with the control and regulation of ongoing behavior^34^,^35^, it is significantly involved in the brain's error processing system^36^. The diminished cortical sensitivity to errors in externalizing pathologies including psychopathy and ADHD^16^,^17^,^18^,^19^,^20^ is correlated with a reduced amplitude of (error-) event related brain potentials (ERP), generated by the ACC^37^, such as the error-related negativity (ERN) which appears in the EEG about 20–150 ms after a committed error also at fronto-central brain sites.

Several studies on brain self-regulation demonstrated that healthy participants can learn to modify cortical activity volitionally with neurofeedback^30^,^38^,^39^. In clinical disorders, neurofeedback can be used to allow communication in completely paralyzed patients^40^,^41^, control a hand prosthesis^42^,^43^, or to normalize the neurophysiological mechanisms underlying specific disorders^44^. Recent research used SCP neurofeedback in disorders with dysregulation of brain excitation like hyperactivation in intractable epilepsy^45^ or frontal hypoactivation such as in ADHD^46^,^47^,^48^ and frontal lobe lesions^49^. Regarding ADHD, Strehl and colleagues^46^ showed diminished attentional problems, reduced impulsivity and improved behavioral symptoms of ADHD after SCP-training, which remained stable at 6 months follow-up. Additionally, improvements in oppositional behavior and physical aggression (indexed by the German Rating Scale for Oppositional Defiant/Conduct Disorders^50^) in children with ADHD after SCP-training were reported^48^.

In psychopathy, treatment studies based on psychophysiological interventions are nonexistent, although neurofeedback was considered as a potential treatment for disinhibited, antisocial and violent behavior^51^. We addressed this issue and investigated if highly criminal psychopaths are able to learn to control their brain activity via neurofeedback, and moreover if psychopathic characteristics like disinhibition, aggression and related behavioral approach tendencies will decrease after neurofeedback training. Furthermore we hypothesized that improved cortical self-regulation skills will improve error processing and attention-related impulsivity on a cortical and behavioral level, resulting in an increased sensitivity to self-inflicted failure.

## Results

### a) SCP-Training

**Figure 1:**
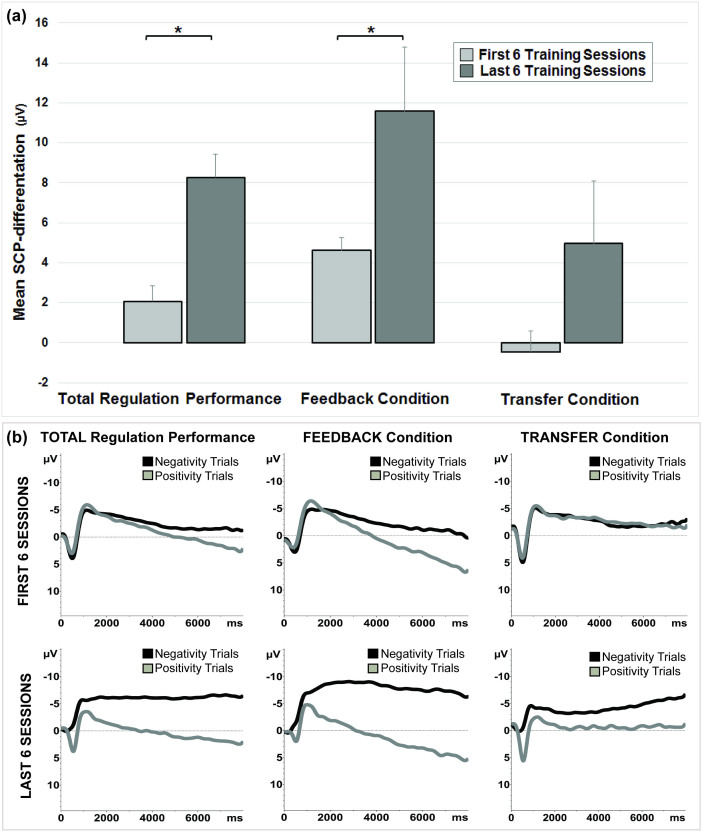
Slow Cortical Potential Neurofeedback. (a). Mean SCP-differentiation of the first 6 and the last 6 training sessions: on the left side for the total regulation performance; and separately for the feedback (in the middle) and for the transfer condition (on the right side). Vertical lines represent the standard error. (b). Upper row: Mean Average of the first 6 training sessions for total (left), feedback (middle) and transfer (right) performance; Electrode FCz. Lower row: Mean Average of the last 6 training sessions for total (left), feedback (middle) and transfer (right) performance; Electrode FCz.

A direct amplitude comparison between the first six and the last six training sessions revealed a significant increase in SCP-differentiation for the total regulation performance (*T*_(5)_ = −4.02, *p* = .005) and for the feedback condition (*T*_(5)_ = −2.01, *p* = .049), but not for the transfer condition (*T*_(5)_ = −1.61, *p* = .084). While the mean SCP-differentiation in feedback was 4.6 µV (*SD* = 1.32 µV) in the first six training sessions, subjects improved their SCP-differentiation to 11.6 µV (*SD* = 7.2 µV) in the last six training sessions. Regarding the transfer condition, the SCP-differentiation was not that pronounced in the beginning (*M* = −0.46 ± 2.3 µV) compared to the feedback condition, but participants learned to produce the correct differentiation at the end of training (*M* = 5.0 ± 7.0 µV) (Figure 1 ab).

Regarding the learning progress over the whole 25 training sessions, regression analysis showed a significant increase of SCP-differentiation for the feedback condition (*R* = .34, *p* = .048) as well as for the transfer condition (*R* = .42, *p* = .018) over time. For additional analysis regarding positive and negative SCP shifts separately see Supplement (“D) Additional Analysis: Differentiating positive and negative SCP shifts”).

### b) Self-Report Measures

**Figure 2:**
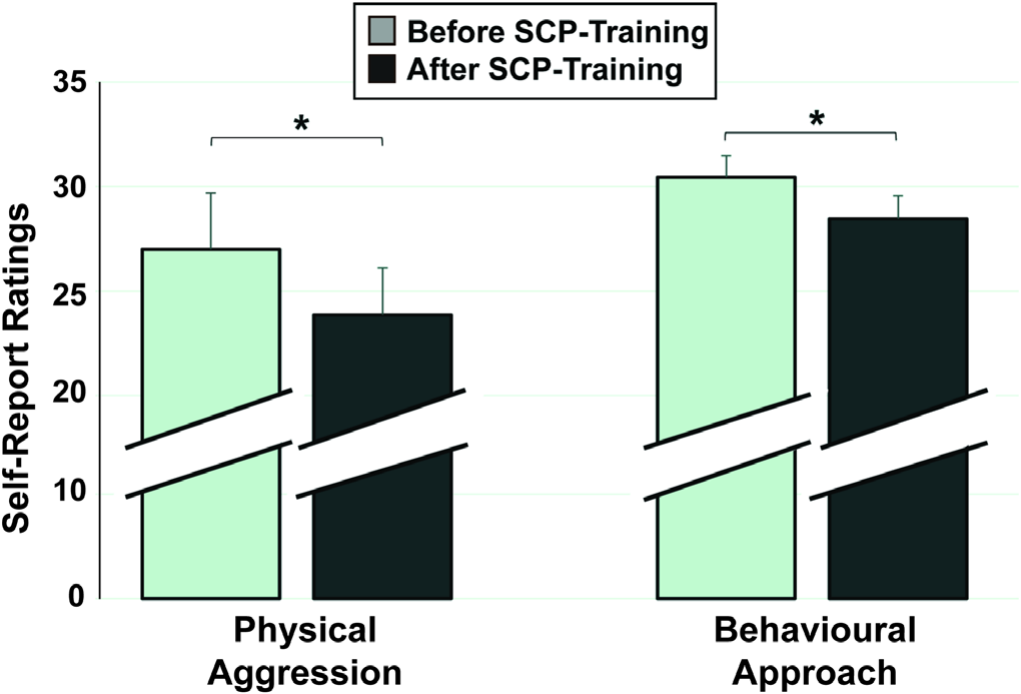
Changes in Self-Reports. Ratings from before to after SCP-Training; Left: Reductions in Physical Aggression (BPAQ); Right: Reductions in Behavioral Approach (BAS). Vertical lines represent the standard error.

A significant reduction in the self-rating of Physical Aggression (BPAQ^52^) was found comparing pre to post SCP-training (*T*_(13)_ = 1.99, *p* = .034) (Figure 2). Furthermore, this change in Physical Aggression (post-pre) was significantly related to the SCP-feedback coefficient (*R* = −.49, *p* = .037): the greater the SCP-differentiation in feedback condition over time, the larger the reductions in Physical Aggression (Table 1).

Secondly, a significant reduction in the self-ratings concerning the behavioral approach system (BAS^53^) was found after SCP-training (*T*_(13)_ = 3.35, *p* = .003), depicted in Figure 2. Similarly, this change in BAS-Total from pre to post training correlated significantly with the SCP-transfer coefficient (*R* = −.47, *p* = .046), indicating higher BAS reductions (post-pre) related to larger SCP-differentiation increases in transfer over time (Table 1).

Although the self-reported reductions in Reactive Aggression and Aggression Inhibition (FAF^54^) did not reach significance from pre to post SCP-training, significant correlations with the SCP-training coefficients (feedback- and/or transfer-coefficients) were observed. The significant correlation between changes in Reactive Aggression (post-pre) and SCP-feedback coefficients (*R* = −.61, *p* = .032) revealed higher reductions in Reactive Aggression related to more pronounced SCP-differentiation in feedback over time. For changes in Aggression Inhibition (post-pre), a significant correlation with SCP-feedback coefficients (*R* = .73, *p* = .008), as well as with SCP-transfer coefficients (*R* = .66, *p* = .020) was found: the higher the increase of SCP-differentiation (in both conditions) over time, the more pronounced the increase in Aggression Inhibition (Table 1).

### c) Flanker Task

Regarding the behavioral analysis, the congruent condition differed significantly from the incongruent condition for incorrect responses (*T*_(13)_ = −2.26, *p* = .020), as well as for correct responses (*T*_(13)_ = 3.76, *p* = .001) before training. A higher number of incorrect (and a lower number of correct) responses in the incongruent condition compared to the congruent condition confirmed the stimulus congruency effect. After SCP-training, the same significant effect was observed for incorrect responses (*T*_(13)_ = −2.71, *p* = .009), but not for correct responses (*T*_(13)_ = 1.30, *p* = .108).

Reaction times were faster for incorrect responses than for correct responses (pre: *T*_(13)_ = 5.01, *p* = .000; post: *T*_(13)_ = 3.12, *p* = .004). Moreover the reaction times were faster for congruent than for incongruent trials (pre: *T*_(13)_ = −4.92, *p* = .000; post: *T*_(13)_ = −7.50, *p* = .000).

Comparing behavioral responses from pre to post SCP-training, the number of correct responses increased significantly (*T*_(13)_ = 1.78, *p* = .048), while Omissions (*T*_(13)_ = 1.84, *p* = .045) and Commission errors (*T*_(13)_ = 1.96, *p* = .035) decreased significantly from pre to post training. For incorrect responses, the reaction times differed significantly (*T*_(13)_ = −3.42, *p* = .003), showing a slowing-down of behavioral reactions after SCP-training (pre: *M* = 342 ± 109.6 ms; post: *M* = 478 ± 92.3 ms) (no significant differences were found from pre to post regarding reaction times for correct responses).

Regarding the ERP analysis, a significant difference in the Error-Related-Negativity amplitude between correct and incorrect responses was observed, showing the ERN amplitude more negative after incorrect responses (error wave) than after correct responses (correct wave) pre (FCz: *T*_(13)_ = −2.92, *p* = .006; Cz: *T*_(13)_ = −4.52, *p* = .000) and post (FCz: *T*_(13)_ = −4.59, *p* = .000; Cz: *T*_(13)_ = −5.05, *p* = .000) SCP-training. This effect was not found for the early Error Positivity (Pe) pre, nor post SCP-training. Only a significant difference in the late Pe amplitude between correct and incorrect responses was found, with the late Pe being more positive after incorrect responses than after correct responses pre (Cz: *T*_(13)_ = 3.90, *p* = .001) and post (Cz: *T*_(13)_ = 3.27, *p* = .003) SCP-training.

**Figure 3:**
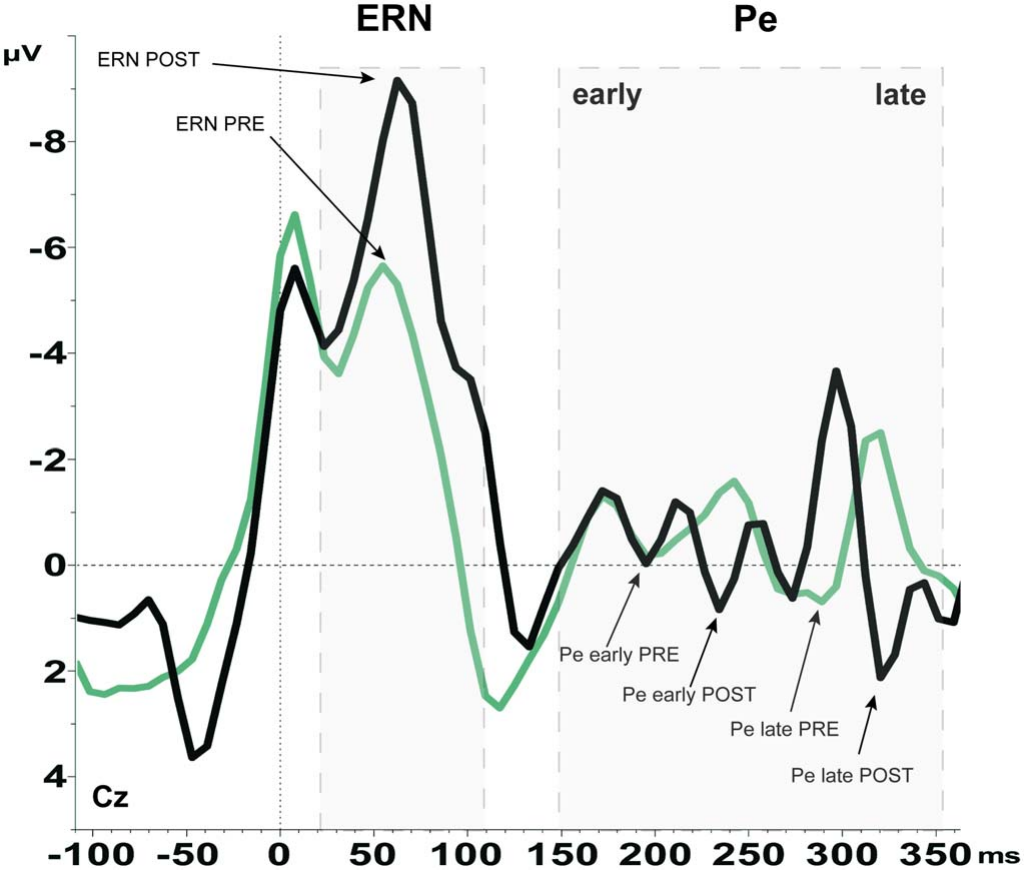
Error-Related-Negativity (ERN) and Error Positivity (Pe). Grand average of Error response-locked waveforms showing the increase in ERN and Pe after (black line) compared to before SCP-Training (gray line). Electrode Cz.

Comparing ERPs from pre to post SCP-training, the effect of time for the Error-ERN from pre to post SCP-training was significant (FCz: *T*_(13)_ = 3.39, *p* = .003; Cz: *T*_(13)_ = 2.88, *p* = .007), demonstrating an increase in the ERN amplitude after SCP-training. Error-Pe analyses revealed, that the late Error Positivity is more pronounced after SCP-training (Cz: *T*_(13)_ = −1.95, *p* = .037), but not the Pe early. The grand averaged error-waveforms (Figure 3) show an increase in ERN from pre (−5.6 µV) to post (−9.2 µV), and for the Pe late from pre (0.7 µV) to post (2.1 µV) SCP-training. Both measurements (pre and post) show comparable latencies for ERN and Pe (ERN: pre = 55 ms, post = 63 ms at FCz; Pe late: pre = 300 ms, post 320 ms at Cz on the grand averaged error-waveforms, depicted in Figure 3).

Based on the difference wave, a significant increase of differentiation between Error-ERN and Correct-ERN from pre to post training was found (FCz: *T*_(13)_ = 4.18, *p* = .000; Cz: *T*_(13)_ = 2.99, *p* = .005); for the Pe only a trend towards an increase was found on the difference wave. Neither ERN, nor Error Pe early/late changed significantly over time on the correct wave.

## Discussion

In the present study, the impact of brain self-regulation on a selected group of severe criminal psychopaths was investigated. For the first time, an extended SCP-neurofeedback-training reveals that offenders scoring high on psychopathy are able to volitionally gain control of their cortical activity. The target brain area for the self-regulation training presented here was the fronto-central region, supposedly involved in self-regulation and behavioral control, accessible to EEG recording for measurements within forensic institutions with high security prison conditions. The physiological function of this SCP-fronto-central system consists of the anticipatory regulation of attentional resources to different cortical target regions critical for regulating behavior through cognitive and affective brain systems^30^. One of the behavioral deficits of psychopathy and ADHD is the lack of preparatory allocation of attentional resources to relevant information and adjusting behavior. Successful SCP self-regulation was investigated in many controlled studies in different groups^30^,^40^,^45^,^46^,^47^,^48^,^49^ and improved executive functioning in most of the studies. The achieved SCP-self-regulation performance of this psychopathic group is comparable to the SCP-self-regulation performance of samples with ADHD, prefrontal lesions and epilepsy, showing an increase in the SCP-differentiation across training sessions, more pronounced in the feedback, than in the transfer condition. Healthy samples achieve the here reported SCP-differentiation much earlier during training (Supplement “(A) SCP-Neurofeedback Research” for comparison).

Here, an exceptional concordance of neurophysiological, subjective-psychological and behavioral modifications in psychopathic patients after SCP-neurofeedback training is reported: the reduced cortical activity in error-processing related to disinhibitory, aggressive, impulsive and psychopathic characteristics^16^,^17^,^18^,^19^,^20^ seems to be compensated (augmented ERN and Pe), associated with an increased cortical sensitivity to failures and errors after SCP-training. This finding is strengthened by the improved behavioral- and aggression- inhibition (less commissions and slower reaction times in the Flanker Task/FAF-scale). Furthermore, the heightened aggression and related behavioral approach tendencies in psychopathy^1^,^3^,^4^ are reduced (BPAQ/BAS-scales) after SCP-training, while attentional switching and focusing is improved, reflected in more correct responses and less omissions in the Flanker Task.

This study aimed to evaluate the possibility of cortical, subjective-psychological and behavioral changes in severe criminal psychopaths after learned SCP-self-control; therefore no causal inferences are possible with a single group pre-post design (Justifications and additional analysis in Supplement “(B) Design of clinical-effect studies in psychopathic offenders”-section). Despite the correlational nature of this study, these results suggest intact learning mechanisms involved in SCP-control and intact underlying brain plasticity in this seemingly change-resistant group.

Although practice effects of repetitive presentation of the Flanker Task cannot be completely excluded, previous test-retest studies showed the Error-Related-Negativity to be remarkably stable and a reliable signal^55^,^56^,^57^. Placebo effects of positive expectancies towards the SCP-neurofeedback training on the subjective measures are certainly possible in a pre-post experimental design but virtually all previous controlled studies using SCP-neurofeedback controlled for placebo effects without any solid evidence for them (see supplement “(B) Design of clinical-effect studies in psychopathic offenders” for a more comprehensive discussion of this issue).

The here demonstrated improvements in neural and attentional flexibility and executive control, together with the behavioral improvements after SCP-neurofeedback training support a hopeful attitude for learned changes in severe criminal psychopaths. The here presented data encourage future studies with larger samples (also to investigate differences between subgroups of psychopathic offenders), more adequate control conditions and control groups and brain self-regulation studies with juvenile inmates or first offenders to track their behavioral developments or potential reoffending over long term.

## Methods

### Experimental Design

To investigate if severe criminal psychopaths are able to gain control of their frontal brain activity and if psychopathic traits such as impulsivity, aggression and excessive behavioral approach will improve after the learned regulation of frontal brain excitation thresholds, we conducted a pre/post-multilevel-cross-validated intensive brain regulation intervention study with a clinically referred sample (Proof-of-Principle). Only an experimental group of criminal participants was trained, aiming to investigate the possibility of brain changes in highly psychopathic offenders after learned SCP-self-control (Justifications of the design in Supplement “(B) Design of clinical-effect studies in psychopathic offenders”-section). SCP-differentiation neurofeedback data of comparable, healthy controls and various patient groups exist for comparison purposes^30^,^40^,^45^,^46^,^47^,^48^,^49^ (Supplement “(A) SCP-Neurofeedback Research”-section).

### Participants

We recruited patients with a long history of criminal records related to serious violent and/or sexual offences (like murder, repeated sexual assault and violent robbery), serving long term sentences in two forensic psychiatric institutions with high security regulations in Germany. Based on the Psychopathy-Checklist-Revised (PCL-R^1^) and on the current clinical status, the final sample consisted of 14 male, adult (mean age: 43.14 ± 11.52 years, all right handed) psychopathic patients with a mean PCL-R score of 30.14 (range: 26–34) (Supplement “(C) Study Subject Recruiting”-section). Patients with an IQ below 80 (on CFT20-R^58^), neurological and medical illnesses or head injuries, as well as patients with major Axis I diagnosis of psychosis, obsessive-compulsive disorder, tics or Tourette- syndrome were excluded. None of the patients received antipsychotic or sedative medication. In agreement with the forensic institutions, offenders received financial compensation for the extensive SCP-training intervention, independent of their performance. Written informed consent was obtained of all participants before being involved in the study. This study was approved by the Ethics Committee of the Medical Faculty of the University of Tübingen according to the Declaration of Helsinki.

### Experimental Procedure and Material

#### a) Slow Cortical Potential Neurofeedback Training

**Figure 4:**
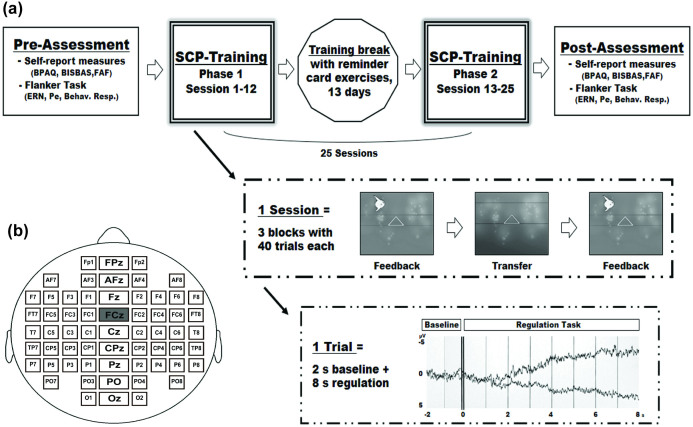
Study Design. (a). Upper row: Procedure and Timing with Pre-Assessment, SCP-Training Phase 1 (12 days), Training break (13 days; Patients were instructed to exercise regulation skills on their own, using a small card showing their preferred training object), SCP-Training Phase 2 (13 days) and Post-Assessment. Middle row: Example of one training session (~60 minutes per day) consisting of three blocks with different conditions: 1.Feedback-> 2. Transfer-> 3. Feedback. Lower row: Example of the time course of an SCP training trial with a 2 sec baseline and the following 8 sec regulation phase is depicted. The upper curve indicates a negative SCP shift, lower curve a positive SCP shift.(b). Location of the EEG feedback site: FCz is depicted (gray).

Slow Cortical Potentials were recorded at FCz (Figure 4b) and fed back to the patients' monitor using graphical objects matched to preferences of the participants (e.g. fish, moon etc.). Each trial started with a triangle, pointing upwards for a required negative SCP-shift, and downwards for a required positive SCP-shift. Participants were instructed to move the object, developing their individual strategy. The instruction emphasized, that muscular (i.e. tension-relaxation) or respiratory strategies disturb self-regulation performance. Successful changes in cortical activity were rewarded with the symbol of a sun after each trial, as the only performance-dependent reinforcement.

All participants underwent 25 SCP-training sessions (each about 60 minutes/day) during a three-month intervention period. The SCP-training consisted of two phases of 13 and 12 training days with a 13 days break. During the break, participants were asked to exercise regulation skills with their individual training strategies using a small reminder card, showing their preferred training object (e.g. fish, moon etc.) to consolidate and transfer successful self-regulation performance.

Each training session consisted of 120 trials, divided into 3 training blocks with different conditions: the first and the last training block were feedback blocks in which the object (e.g. a fish) moved from left to right over the screen to provide feedback according to the SCP activity. The middle training block was designed as a transfer block in which the object (e.g. the fish) did not appear on the subjects' monitor - only a blue screen was presented during the SCP regulation task without any feedback of brain activity to the person (except the symbol of the sun after successful regulation trials). One single training trial comprised a 2 second baseline followed by an 8 second active regulation phase. During the Inter-Trial-Interval (ITI; varying between 1 and 2 sec, randomized) an empty blue screen or -after successful regulation trials, a reinforcement screen (with a symbol of a sun)- was presented. Required negativity (50% in the first; 80% in the second training phase) and required positivity (50% in the first; 20% in the second training phase) were presented in random order in accordance with established neurofeedback training protocols in ADHD. Every SCP-training session started with an eye movement calibration task, which was used for an online eye movement artefact correction during the training to control und minimize influences caused by eye movements^59^. An overview of the study design, an example of one session (including feedback and transfer blocks) and one trial are given in Figure 4a, Figure 4b depicts the location of the EEG feedback site: FCz.

#### b) Self-Report Measures

All questionnaires were handed to the participants before and after the SCP-training (Figure 4). Aggression was assessed using the German questionnaire for the assessment of aggressiveness factors (FAF^54^) and the Buss-Perry-Aggression Questionnaire (BPAQ^52^). The Behavior-Inhibition/Behavior-Activation System Questionnaire (BIS/BAS^53^) served as an index for behavioral approach (BAS), sensitive to reward and associated with aggression, anger and impulsivity^3^,^4^,^53^ and behavioral inhibition (BIS), sensitive to punishment and related to anxiety^53^.

#### c) Flanker Task

To investigate impulsivity, attention, error processing as well as behavioral inhibition, a modified letter version of the Eriksen Flanker Task^60^ was applied before and after SCP-training (Figure 4). In the letter flanker task, participants had to respond to the center letter of a 5-letter string with a right hand button press for the target ‘S' and a left hand button press for the target ‘H' (button-letter arrangement was counterbalanced), while they should not press any button if ‘X' was the center letter (‘non-target trials'). The letter strings of the ‘target trials' were either congruent (HHHHH or SSSSS) or incongruent (SSHSS or HHSHH), as well as the ‘non-target trials' (congruent: XXXXX, incongruent: SSXSS or HHXHH), with the incongruent condition characterized by a higher degree of difficulty (stimulus congruency effect). After a red fixation cross on white background, the black letter strings appeared on the screen for 150 ms followed by an inter-trial interval of 1000 ms. In total 400 trials including 20% non-target trials (both with the same amount of congruent and incongruent trials) were presented.

To investigate cortical activity related to error-processing, two Event-Related Potentials (ERPs) were analyzed: the Error Related Negativity (ERN), reflecting early stages in error monitoring without conscious awareness of the errors and the following Error Positivity (Pe), associated with later stages of error processing with conscious awareness of the errors^61^. On the behavioral level, the number of correct and incorrect responses, omissions and commissions, as well as reaction times were recorded. Written instructions were given and test comprehension was evaluated with exercise trials before starting the computerized task.

### EEG-Recording and Data Processing

EEG measurements were collected using a 22-channel Theraprax Q-EEG-System (NeuroConn GmbH, Illmenau, Germany). During SCP-training recordings the electrode FCz was used, while Fz, FCz, Cz and Pz were recorded during the Eriksen Flanker Task. The left mastoid was used as reference and the right mastoid as ground. Electrooculography was measured by placing electrodes above and below the left eye for blinks and vertical eye movements and at the outer canthi for horizontal eye movements. Electrode impedances were kept below 3kOhm throughout the study. The signals were recorded with a sampling rate of 128 Hz and with a 40 Hz low pass filter. EEG artifacts were detected automatically during the SCP-training recordings by the Theraprax System with a movement artifact correction or the trial was cancelled and repeated. Data from the eye movement calibration task before each training session was used for online artefact correction during the training to control und minimize influences caused by eye movements^43^. All EEG data were further processed using Brain Vision Analyzer Professional 2.01 (BrainProducts GmbH, Gilching, Germany). The signal was 50 Hz notch filtered and EOG artifacts were additionally corrected offline, based on Gratton et al.^62^.

From artifact-free *SCP-training recordings*, data between 0.1–2 Hz was used and baseline corrected, relative to -2000 ms pre-regulation phase. For each participant, the SCP-recordings were separately averaged for the two conditions (feedback and transfer) and tasks (required positivity and negativity) for each session. Only the last four seconds of each regulation trial were used for SCP-amplitudes to exclude influences of early ERPs on the SCP data.

Regarding the physiological *Flanker-Task analysis*, a 1–30 Hz filter was used for all midline positions. Artifact-free EEG recordings for non-target- and target-trials were time-locked to response onset and averaged separately for correct and incorrect responses for each participant relative to a -500 ms pre-response baseline. ERN was defined as the most negative peak in the 20–150 ms period following response onset at fronto-central sites. Pe was split into two components: the early Pe was defined as the most positive peak between 150-260 ms, while the late Pe was calculated for 261–350 ms post-response at Cz. ERN and Pe peak amplitudes were considered based on the correct-, the incorrect- (error) and the difference-waveform (errorwave minus correctwave) of each participant pre and post SCP-training.

### Statistical Analysis

At first, the mean SCP-differentiation (mean µV-amplitude difference between required positivity and negativity) was calculated for each participant and for each of the 25 sessions, separately for feedback- and for transfer-condition. In order to examine the group performance of the SCP-training, paired sample t-tests were used to compare the average SCP-amplitude of the first six and the last six training sessions, reflecting the total regulation performance (across all trials) and separately for feedback- and transfer-condition. For a detailed examination of the learning course of regulation skills over time, linear regression analyses were performed using the average SCP-differentiation as independent and time (25 sessions) as the dependent variable. For analyses regarding relationships between individual training performance and changes in self-report measures (post-pre), an individual learning-indicator, the regression coefficient (non-standardized ß-value of the linear regression for each participant, separately for both conditions) was used.

Pre-post training comparisons regarding changes in questionnaire scores were performed using paired sample t-tests. In order to test for an association between these changes in time (post-pre) and the training performance, Pearson correlations were performed.

For the analysis of the Flanker Task, paired sample t-tests were used to detect changes in behavioral responses (number of correct/erroneous responses for both conditions and reaction times) as well as to detect changes in peak values for ERN and Pe (amplitude and latency based on the correct-, erroneous- and difference wave).

Although no violation of the normal distribution was found in the data, non-parametric analysis were additionally performed, but yielded the same results and were therefore omitted in the present text. Bonferroni correction was applied for multiple comparisons (which resulted in a corrected α of 0.025 for BIS/BAS-, and a corrected α of 0.01 for FAF-questionnaire pre/post analyses). Since there were a priori directed hypotheses, one tailed p-values were used for all statistical analyses.

## Author Contributions

L.K., R.V. and N.B. conceived and designed the study, L.K., B.B. and H.E. conducted the training and experiments, L.K. and R.V. analyzed the data, L.K., R.V., B.B., P.T., U.S. and N.B. interpreted the data, L.K. wrote the paper, R.V., N.B., B.B., U.S., P.T. and H.E. edited and revised the paper. All authors contributed to the preparation of the paper and approved the final manuscript.

## Supplementary Material

Supplementary Information Supplement: Brain self-regulation in criminal psychopaths

## Figures and Tables

**Table 1:** Correlations between changes in self-reports (post-pre) and SCP-training coefficients (feedback and transfer)

	Feedback coefficient	Transfer coefficient
**Physical Aggression**	−.49*	.01
**Behavioral Approach**	−.27	−.47*
**Reactive Aggression**	−.61*	−.53
**Aggression Inhibition**	.73**	.66*

Each cell consists of Pearson correlation coefficients. *<0.05; ** p<0.01.
