# Supplementary material for: Brain self-regulation in criminal psychopaths
Source: Sci Rep. 2015 Mar 24;5:9426. doi: 10.1038/srep09426 (PMC4371087; doi:10.1038/srep09426)
Supplement: Supplementary Information — Supplement: Brain self-regulation in criminal psychopaths [file srep09426-s1.pdf]

Brain self-regulation in criminal psychopaths

Lilian Konicar, Ralf Veit, Hedwig Eisenbarth, Beatrix Barth, Paolo Tonin, Ute Strehl and Niels Birbaumer

SUPPLEMENTARY MATERIALS

(A)	SCP-Neurofeedback Research	2
(B)	Design of clinical-effect studies in psychopathic offenders	3
(C)	Study Subject Recruiting	5
(D)	Additional Analysis: Differentiating positive and negative SCP	7
	References	9

(A) SCP-Neurofeedback Research

The differentiation between learned cortical negativity and positivity of the presented psychopathic patients in this study is in line with the performance of samples with comparable behavioral and cortical abnormalities: in subjects with ADHD, Strehl and colleagues[1] showed SCP-differentiation amplitudes of ~4μV in the feedback- and ~ -1.3μV in the transfer condition at the beginning of the training (session 2+3), while the SCP-differentiation increased to ~7.3μV in the feedback- and to ~ -2.8μV in the transfer condition at the end of the training (session 29+30). Similar differentiation amplitudes at the end of the training were found also in an early study with children with attentive problems (~10μV in feedback; ~-1μV in transfer)[2].

In our psychopathic sample we found comparable regulation changes, but more pronounced increases from the beginning until the end of the training in the feedback condition, as well as in the transfer condition. The slightly smaller differentiation performances in the studies of Strehl and colleagues[1] and Rockstroh and colleagues[2] might be due to the generally smaller differentiation in children compared to adults[3]. Regarding healthy adults, SCP-feedback differentiation amplitudes around 12μV were usually already found after three to four sessions of training[3], which matches the SCP-differentiation achievements of our sample at the end of the training (even though the reported SCP-differentiation amplitudes in the transfer condition were substantially larger in healthy subjects compared to our forensic sample). The SCP regulation abilities of patients with frontal lobe damage are much smaller -compared to healthy samples- in both conditions[4] (~5μV in feedback; ~ -2μV in transfer after two training sessions), similar to our participants and ADHD samples at the beginning of the training. In intractable epilepsy, the SCP neurofeedback group achieved substantial differentiation of SCPs after 30 sessions, but not the alpha-activity neurofeedback group, with more pronounced SCP-differentiation in healthy compared to epileptic patients[5] (for an SCP

research overview see[3]). Patients with severe epilepsy show no SCP self-regulation in the first training sessions comparable to the psychopathic group of this study.

Interestingly, the same pattern as in the ADHD-study of Strehl and colleagues[1] was found in our psychopathic sample, with difficulties to produce the correct polarities at the beginning of the training, especially in the transfer condition; however, our psychopathic patients learned to produce the correct polarities at the end of the training and could increase the SCP-differentiation also in the transfer condition. In line with many SCP-studies in clinical samples [e.g. 1,6,7], we found smaller SCP differentiations in the transfer compared to the feedback condition.

Based on the results of a meta-analysis, including studies showing the superiority of neurofeedback training compared to non-treatment[e.g. 8] or other treatments[e.g. 9], Arns and colleagues[10] concluded -with respect to ADHD- that neurofeedback can be considered as an efficacious and specific treatment regarding inattention, impulsivity and hyperactivity.

(B) Design of clinical-effect studies in psychopathic offenders

From a methodological point of view, a double blind controlled design is warranted to prove clinical effectivity. In such a design matched participants would have received (a) a psychological/behavioral treatment or (b) psychophysiological feedback based on another EEG parameter, like alpha activity; or sham SCP-feedback or feedback from electromyographical activity, as a control strategy. The aim of this study was to investigate the self-regulation abilities and related behavioral outcomes in highly psychopathic patients, and not the comparison of neurofeedback with other similar treatments, because the efficacy and performance of neurofeedback was repeatedly shown in other studies and different samples: see (A) SCP-Neurofeedback Research in Supplementary Material. Still, comparing the training outcome measures with a non-treatment group or a control group (a,b), would

have required at least an age-, psychopathy- and offence- matched control group, which is not available in the German forensic hospitals (note that our sample already consisted of offenders with high psychopathy scores and extreme crimes, exceeding markedly the proposed cut-off score for German[11] and European[12] psychopathic samples). The comparison between our highly psychopathic group with severe and multiple offences and a group consisting of participants with less severe offences or low scores on the PCL-R might have revealed specific differences, but cannot be regarded as a matched control group. In addition, blinding of therapists in self-regulation treatment is impossible, because patients and therapists are continuously informed of the achieved brain changes, which leads to conscious or subliminal perception of treatment progress and therefore uncontrolled placebo responses. Placebo effects of SCP-training were excluded in most of the previous studies with healthy and psychopathological samples and different types of control procedures[2]. The physiological nature of the task, the extensive training time and the neurobiological measure limits the possibilities of placebo response, but certainly cannot exclude them completely.

Whether the modification of the cognitive and emotional behavior measured in this sample is sufficient for the compensation of the emotional and social deficits in psychopathy, is an empirical question and needs larger sample sizes - a significant limitation of the present study. We split our sample at the median of the Total PCL-R score in to 'very high' and 'medium to high' psychopathy, but did not find any significant differences regarding the SCP-regulation performance, neither for the feedback ($T_{(13)}$=.283, p=.782), nor for the transfer ($T_{(13)}$=.247, p=.809) condition between the two groups. Besides the expected, highly significant correlation between the regulation performance in the two conditions (feedback, transfer) (R=.784, p=.001), correlational analysis revealed no significant relationships between the regulation performance and the PCL-R score, neither for feedback (R=-.369, p=.195), nor for transfer (R=-.348, p=.223). The result, that we did not find any significant correlations with the PCL-R scores, can be due to the lack of variation of PCL-R scores as a consequence of the

extreme psychopathy values of this group ("plafond effect"). Accordingly, these findings point towards an ability to learn brain self-regulation in high and less-high psychopathic offenders.

Besides questionable validity of staff observations inside prisons or high security forensic units (or behavioral tests inside those institutions), the evaluation of behavioral changes and the internalization of proper social rules of conduct after the treatment of psychopathic patients, is a difficult task. Releasing successfully trained/ treated criminal patients and tracking their aggressive behavior or recidivism is out of question, because of ethical reasons and public safety. Only long-term follow ups after legal release from prison or forensic unit may provide an answer to the generalization problem. Most of the participants of our sample committed offences, which are usually punished with lifelong imprisonment or ordered to undergo indefinite time treatment in high security forensic psychiatry units.

C) Study Subject Recruiting

Only participants with a PCL-R score of ≥ 26 were accepted in the study, which is above the proposed cut-off score for the German and European samples[11,12.].

Only patients according to section §63 or §66 of the German Criminal Law were recruited for this study.

Section 63: Placement in a Psychiatric Hospital

If someone committed an unlawful act and at the time lacked capacity to be adjudged guilty (Section 20) or was in a state of diminished capacity (Section 21), the court shall order placement in a psychiatric hospital if a comprehensive evaluation of the perpetrator and his act reveals that, as a result of his condition serious unlawful acts can be expected of him and he therefore presents a danger to the general public.

Section 20: **Lack of Capacity to be Adjudged Guilty due to Emotional Disorders**

Whoever upon commission of the act is incapable of appreciating the wrongfulness of the act or acting in accordance with such appreciation due to a pathological emotional disorder, profound consciousness disorder, mental defect or any other serious emotional abnormality, acts without guilt.

Section 21: **Diminished Capacity to be Adjudged Guilty**

If the capacity of the perpetrator to appreciate the wrongfulness of the act or to act in accordance with such appreciation is substantially diminished upon commission of the act due to one of the reasons indicated in Section 20, then the punishment may be mitigated pursuant to Section 49 subsection (1).

Section 66 Placement in Preventive Detention

(1) If someone is sentenced for an intentional crime to a fixed term of imprisonment of at least two years, then the court shall order preventive detention collateral to the punishment, if:

(2) If someone has committed three intentional crimes for which he incurred, respectively, imprisonment for at least one year, and if he is sentenced to a fixed term of imprisonment of at least three years for one or more of these acts, then the court may under the provision indicated in subsection (1), no. 3, order preventive detention collateral to the punishment even without a prior sentence or deprivation of liberty (subsection (1), nos. 1 and 2).

[Subsections (3), (4) and further information available online: http://www.iuscomp.org/gla/statutes/StGB.htm]

D) Additional Analysis: Differentiating positive and negative SCP shifts

Additional analysis, differentiating the two polarities into negative SCP shifts and positive SCP shifts are summarized in the table d1 and d2.

d1) Comparison: First 6 Training Sessions – Last 6 Training Sessions

A comparison of the amplitude of negative SCP shifts of the feedback condition reveals a significant increase ($T_{(5)} = 2.233$, $p = .038$) from -1.35μV in the first six training sessions to -8.89 μV in the last six training sessions, as shown in Table 2. A comparison of the first six SCP-sessions to the last six SCP sessions separately for positive SCP shift of the feedback condition, as well as for the negative and positive SCP shifts of the transfer condition did not show significant changes.

Table 2: Negative and Positive SCP changes in Feedback and Transfer Condition of the first 6 and last 6 Training Sessions.

	**Feedback Negative SCPs**	**Feedback Positive SCPs**	**Transfer Negative SCPs**	**Transfer Positive SCPs**
**First 6 SCP-sessions Mean *(sd)***	-1.35μV *(4.4μV)*	3.34μV *(4.70μV)*	-1.37μV *(2.55μV)*	-1.83μV *(2.29μV)*
**Last 6 SCP-sessions Mean *(sd)***	-8.89μV *(9.02μV)*	2.70μV *(5.45μV)*	-3.16μV *(5.29μV)*	1.80μV *(2.46μV)*
$T_{(5)}$	2.233	.214	.712	-1.397
P	*.038**	0.419	.254	.111

<u>Tab. 2:</u> In the first two lines, every cell consists of the *Mean* μV for the first, respectively the last 6 training sessions, and the standard deviation (*sd*) in italic. The related *T*-value, including the degrees of freedom in parentheses and subscripted, as well as the *P*-value is depicted in line three and four.

d2) Regression Analysis over 25 SCP-Training Sessions

Regarding the learning process over the whole 25 SCP-sessions, regression analysis showed a significant increase in negative SCP-shifts in the feedback condition (R = -.347, p = .045), but not in positive SCPs in the feedback condition, as depicted in Table 3. Regarding the transfer condition, a trend for an increase in positive SCP amplitude was found (R = .320, p = .059), while the amplitude of negative SCP increased only minimal over time.

Table 3: Regression Analysis for Negative and Positive SCPs in Feedback and Transfer Condition.

	**Feedback Negative SCPs**	**Feedback Positive SCPs**	**Transfer Negative SCPs**	**Transfer Positive SCPs**
R	-,347	-,143	-,068	,320
P	*.045**	.247	.374	*.059*

Tab. 3: In the first lines the correlation coefficient R is presented. The second line depicts the P-value of the regression.

For all polarity-sensitive analysis which are not based on SCP differentiation, but on separate analysis differentiating negative and positive SCP shifts, the setup of the training-protocol including its different phases (first phases 50% : 50% negativity/positivity; second phase 80% : 20%) has to be considered.

REFERENCES

1. Strehl, U. et al. Self-regulation of slow cortical potentials: A new treatment for children with attention-deficit/hyperactivity disorder. *Pediatrics* **118**, e1530- e1540 (2006).
2. Rockstroh, B., Elbert, T., Lutzenberger, W. & Birbaumer, N. The effects of slow cortical potentials on response speed, *Psychophysiology* **19**, 211-17 (1982).
3. Rockstroh, B., Elbert, T., Canavan, A., Lutzenberger, W. & Birbaumer, N. *Slow Cortical Potentials and Behaviour* (Urban and Schwarzenberg, 1989).
4. Lutzenberger, W. et al. in *Motivation, motor and sensory processes of the brain. Electrical potentials, behavior and clinical use* (eds. Kornhuber, H.H. et al) 427-430 (Elsevier, 1980).
5. Elbert, T., Rockstroh, B., Birbaumer, N., Canavan, A., & von Billow, I. in *Biobehavioral Self-Regulation and Health (eds.* Carlson J.G. *et al.*) 65-94 (Plenum Press, 1990).
6. Kotchoubey, B. et al. Self-regulation of slow cortical potentials in epilepsy: a retrial with analysis of influencing factors. *Epilepsy Res.* **25,** 269–76 (1996).
7. Kotchoubey, B. et al. Modification of slow cortical potentials in patients with refractory epilepsy. *Epilepsia* **42**, 406-16 (2001).
8. Heinrich, H., Gevensleben, H., Freisleder, F.J., Moll, G.H. & Rothenberger, A. Training of slow cortical potentials in attention-deficit/ hyperactivity disorder: evidence for positive behavioral and neurophysiological effects. *Biol. Psychiatry* **55**, 772-75 (2004).
9. Gevensleben, H. et al. Distinct EEG effects related to neurofeedback training in children with ADHD: a randomized controlled trial. *Int. J. Psychophysiol.* **74**, 149-57 (2009).
10. Arns, M., de Ridder, S., Strehl, U., Breteler, S. & Coenen, A. Efficacy of neurofeedback treatment in ADHD: the effects on inattention, impulsivity and hyperactivity: a meta-analysis, *Clin. EEG Neurosci.* **40**, 180-9 (2009).
11. Mokros, A. et al. Normative Data for the Psychopathy Checklist–Revised in German-Speaking Countries: A Meta-Analysis. *Crim. Justice Behav.* **40**, 1397-412 (2013).
12. Cooke, D. J., Hart, S. D. & Michie, C. Cross-national differences in the assessment of psychopathy: do they reflect variations in raters' perceptions of symptoms? *Psychol. Assess.* **16**, 335–39 (2004).
